# Early Predictors of the Increase in Perihematomal Edema Volume After Intracerebral Hemorrhage: A Retrospective Analysis From the Risa-MIS-ICH Study

**DOI:** 10.3389/fneur.2021.700166

**Published:** 2021-07-27

**Authors:** Gengzhao Ye, Shuna Huang, Renlong Chen, Yan Zheng, Wei Huang, Zhuyu Gao, Lueming Cai, Mingpei Zhao, Ke Ma, Qiu He, Fuxin Lin, Yuanxiang Lin, Dengliang Wang, Wenhua Fang, Dezhi Kang, Xiyue Wu

**Affiliations:** ^1^Department of Neurosurgery, The First Affiliated Hospital of Fujian Medical University, Fuzhou, China; ^2^Department of Clinical Research and Translation Center, The First Affiliated Hospital of Fujian Medical University, Fuzhou, China

**Keywords:** intracerebral hemorrhage, perihematomal edema, black hole sign, PHE expansion, predictors

## Abstract

**Background and Purpose:** Perihematomal edema (PHE) is associated with poor functional outcomes after intracerebral hemorrhage (ICH). Early identification of risk factors associated with PHE growth may allow for targeted therapeutic interventions.

**Methods:** We used data contained in the risk stratification and minimally invasive surgery in acute intracerebral hemorrhage (Risa-MIS-ICH) patients: a prospective multicenter cohort study. Patients' clinical, laboratory, and radiological data within 24 h of admission were obtained from their medical records. The absolute increase in PHE volume from baseline to day 3 was defined as iPHE volume. Poor outcome was defined as modified Rankin Scale (mRS) of 4 to 6 at 90 days. Binary logistic regression was used to assess the relationship between iPHE volume and poor outcome. The receiver operating characteristic curve was used to find the best cutoff. Linear regression was used to identify variables associated with iPHE volume (ClinicalTrials.gov Identifier: NCT03862729).

**Results:** One hundred ninety-seven patients were included in this study. iPHE volume was significantly associated with poor outcome [*P* = 0.003, odds ratio (OR) 1.049, 95% confidence interval (CI) 1.016–1.082] after adjustment for hematoma volume. The best cutoff point of iPHE volume was 7.98 mL with a specificity of 71.4% and a sensitivity of 47.5%. Diabetes mellitus (*P* = 0.043, β = 7.66 95% CI 0.26–15.07), black hole sign (*P* = 0.002, β = 18.93 95% CI 6.84–31.02), and initial ICH volume (*P* = 0.018, β = 0.20 95% CI 0.03–0.37) were significantly associated with iPHE volume. After adjusting for hematoma expansion, the black hole sign could still independently predict the increase of PHE (*P* < 0.001, β = 21.62 95% CI 10.10–33.15).

**Conclusions:** An increase of PHE volume >7.98 mL from baseline to day 3 may lead to poor outcome. Patients with diabetes mellitus, black hole sign, and large initial hematoma volume result in more PHE growth, which should garner attention in the treatment.

## Introduction

Spontaneous intracerebral hemorrhage (ICH) is a severe form of stroke and accounts for 10–20% cerebrovascular diseases (1). In the past, the treatment of ICH mainly focused on primary injury and inhibiting hematoma growth. However, these treatment strategies do not seem to have achieved satisfactory results (2, 3). More and more attention has been paid to the secondary damage after cerebral hemorrhage.

After the initial injury caused by brain tissue disruption and mass effect of the hematoma, the activation of a coagulation cascade reaction, inflammatory cell infiltration, and the product of erythrocyte rupture triggers a series of secondary harmful events, eventually leading to the formation of perihematomal edema (PHE) (2, 4–6). A growing body of research shows that PHE was associated with poor functional outcomes after ICH (7–13), and treatment strategies to attenuate PHE are likely to improve patient outcomes (14). PHE progresses fastest in the first 2 to 3 days (4), which leaves a therapeutic time window. Therefore, early identification of risk factors associated with PHE growth may allow for the implementation of a more aggressive treatment strategy.

In this study, we aimed to explore the relationship between PHE and poor outcome and, more importantly, to find out the predictors of PHE growth.

## Methods

### Study Design and Population

We used data contained in the risk stratification and minimally invasive surgery in acute intracerebral hemorrhage (Risa-MIS-ICH) study patients: a prospective multicenter cohort study. The study consists of two parts: The first part is to conduct a multicenter retrospective analysis of acute ICH patients from 33 centers in China to create a predictive model of ICH growth based on clinical, blood, genetic, imaging, and pharmacological factors; the second part is to validate the efficacy of the minimally invasive surgery, including stereotactic thrombolysis and endoscopic surgery, in eligible patients with high risk of hemorrhage growth according to the first part results in a prospective multicenter cohort study. The retrospective data were used in this study. The inclusion criteria for patient enrollment were as follows: (1) confirmation of ICH by cerebral imaging within 24 h of onset; (2) no cerebral herniation (defined as a shift of cerebral tissue from its normal location into an adjacent space that is life-threatening and requires prompt surgical intervention) at admission; (3) Glasgow Coma Scale (GCS) score > 5 at admission. Exclusion criteria were (1) secondary ICH due to aneurysm, vascular malformation, hemorrhagic infarction, tumor, or coagulation disorders; (2) occurrence of infratentorial hemorrhage; (3) evidence of pregnancy; (4) patients suffering from tumor, ischemic heart disease, kidney, hepatic insufficiency, or any disease with life expectancy <3 months. Informed consent was taken from all patients. This study was registered with ClinicalTrials.gov (No. NCT03862729).

### Data Collection and Outcome Evaluation

Data were retrieved based on electric medical records, including demographic character, initial symptoms, vital signs, medical history, laboratory data and imaging characteristics. Hematoma and PHE volumes were measured following the method of Gusdon et al. (15) using semiautomated planimetry with a three-dimensional slicer. Similar to the previous study (9), the absolute increase in PHE volume from baseline to the third day was defined as iPHE volume. The presence of black hole sign, swirl sign, island sign, and hematoma sedimentation level were used as imaging predictors for early hematoma growth (16–20). We attempted to evaluate their roles in predicting PHE growth; the imaging interpretation criteria used in this study are the same as the previous studies (16–20). Shape and density categorical scales were likewise defined in a previous study (20). Hematoma expansion was defined as an increase in ICH volume between baseline and repeat imaging of more than 6 mL or more than 33% (21). The imaging evaluation was performed by two experienced neuroradiologists who were unaware of the other variables and outcomes. Data collection of laboratory results used the first-time examination at admission (within 24 h after admission). Follow-up data were acquired by medical records, telephone interviews, or outpatient clinics. Poor outcome was defined as modified Rankin Scale (mRS) of 4 to 6 at 90 days.

### Statistical Analysis

Statistical analyses were performed using the SPSS software package (version 25.0, IBM Corporation). Baseline demographic and clinical characteristics were descriptively summarized as mean ± standard deviation (SD) or median [interquartile range (IQR)] for continuous variables and as number (%) for categorical variables. The demographics and ICH characteristics were compared between included and excluded patients using Chi-square test, Student's *t-*test, or the Mann–Whitney *U*-test as appropriate. Binary logistic regression and restricted cubic splines with three knots were used to assess the relationship between iPHE volume and poor outcome. The receiver operating characteristic (ROC) curve was used to establish the best cutoff point of iPHE volume for predicting poor outcome. Linear regression was used to identify variables associated with iPHE volume. Factors contributing significantly in univariate analysis or showing a trend toward *P* < 0.10 were entered into a multivariable model. Two-sided *P*-values < 0.05 was considered significant.

## Results

### Patient Characteristics

**Figure 1:**
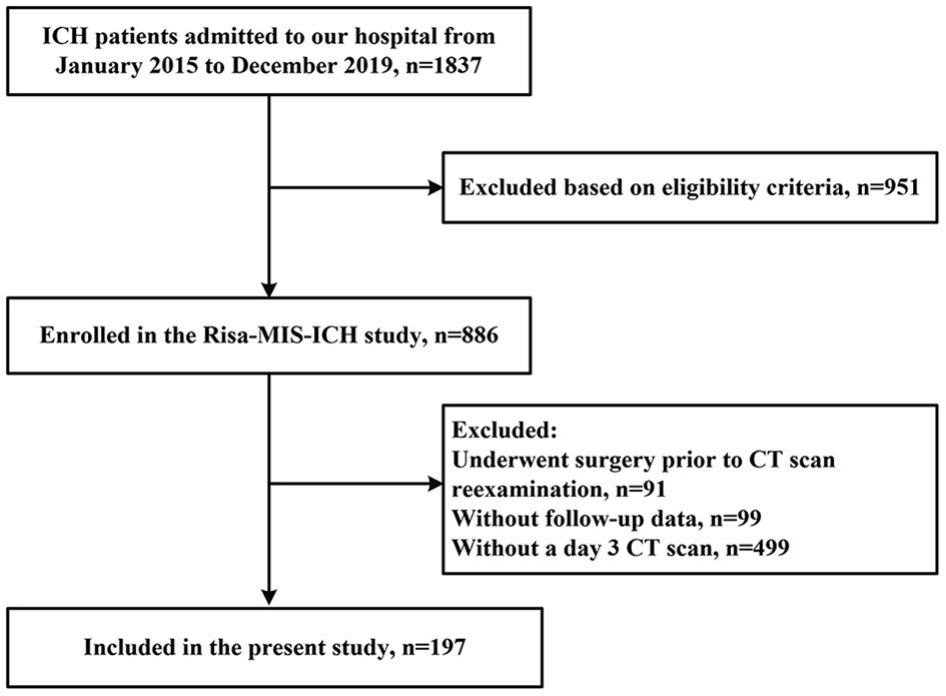
Flow chart of patient selection.

Of a total of 886 patients with ICH entered into our database, 689 patients were excluded due to incomplete clinical data, and 197 patients were included in this study (the enrollment flow chart of the study population is shown in Figure 1). There were more males (*n* = 134, 68%) than females (*n* = 63, 32%) enrolled. The average age was 59.6 (±12.9) years. Twenty patients (10.1%) had a history of diabetes mellitus. A total of 52 (26.4%) patients presented with lobar hemorrhage and 145 (73.6%) with deep hemorrhage. Median ICH volume on admission was 12.7 mL (IQR 5.8–20.9), and median PHE volume at baseline was 9.8 mL (IQR 4.4–19.6). Median GCS on admission was 14 (IQR 12–15), median mRS at 90 days was 4 (IQR 3 to 5). There were no significant differences between the two cohorts in age, sex, hematoma volume, diabetes history, black hole sign, and other major factors. However, patients included had a relatively higher admission GCS score due to the exclusion of patients undergoing surgery at early stage. Demographic, clinical, and radiological characteristics are shown in Table 1.

**Table 1:** Demographics and ICH characteristics of included and excluded patients.

**Variables**	**Included patients *n* = 197**	**Excluded patients *n* = 689**	***P*-value**
Age (year), mean (SD)	59.6 (±12.9)	60.0 (±12.8)	0.672
Gender			
Male, *n* (%)	134 (68.0%)	485 (70.4%)	0.261
Female, *n* (%)	63 (32.0%)	204 (29.6%)	0.261
Admission SBP (mmHg), median (IQR)	161 (140–175)	160 (142–178)	0.468
Admission DBP (mmHg), median (IQR)	90 (81–100)	92 (80–102)	0.425
Admission GCS, median (IQR)	14 (12–15)	14 (10–15)	0.017
Diabetes mellitus, *n* (%)	20 (10.1%)	84 (12.2%)	0.231
Hypertension, *n* (%)	138 (70.1%)	495 (71.2%)	0.623
Admission serum glucose (mmol/L), median (IQR)	5.8 (5.0–6.0)	6.5 (5.4–8.3)	<0.001
Onset to admission scan (h), median (IQR)	7.4 (4.1–16.4)	5.8 (3.1–12.0)	0.014
Hematoma volume at baseline (mL), median (IQR)	12.7 (5.8–20.9)	13 (5.5–30.5)	0.275
PHE at baseline (mL), median (IQR)	9.8 (4.4–19.6)	10.6 (4.4–20.8)	0.658
Lobar location of ICH, n (%)	52 (26.4%)	174 (25.3%)	0.746
Deep location of ICH, n (%)	145 (73.6%)	515 (74.7%)	0.746
Presence of IVH, n (%)	62 (31.3%)	244 (35.4%)	0.305
Shape categorical scales, median (IQR)	2 (1–2)	2 (1–3)	0.227
Density categorical scales, median (IQR)	1 (1–2)	1 (1–2)	0.344
Black hole sign, *n* (%)	7 (3.5%)	48(7.0%)	0.080
Swirl sign, *n* (%)	10 (5.1%)	30 (4.4%)	0.667
Island sign, *n* (%)	7 (3.5%)	64 (9.3%)	0.009
Hematoma sedimentation level, *n* (%)	2 (1.0%)	12 (1.7%)	0.426
mRS score >3 at 90d, *n* (%)	99 (50.3%)	Incomplete data.	-

*GCS, Glasgow Coma Scale; IVH, intraventricular hemorrhage; SD, standard deviation; IQR, interquartile range; SBP, systolic blood pressure; DBP, diastolic blood pressure; ICH, intracerebral hemorrhage; mRS, modified Rankin Scale; PHE, perihematomal edema*.

### Correlation Between iPHE Volume and Poor Outcome

**Figure 2:**
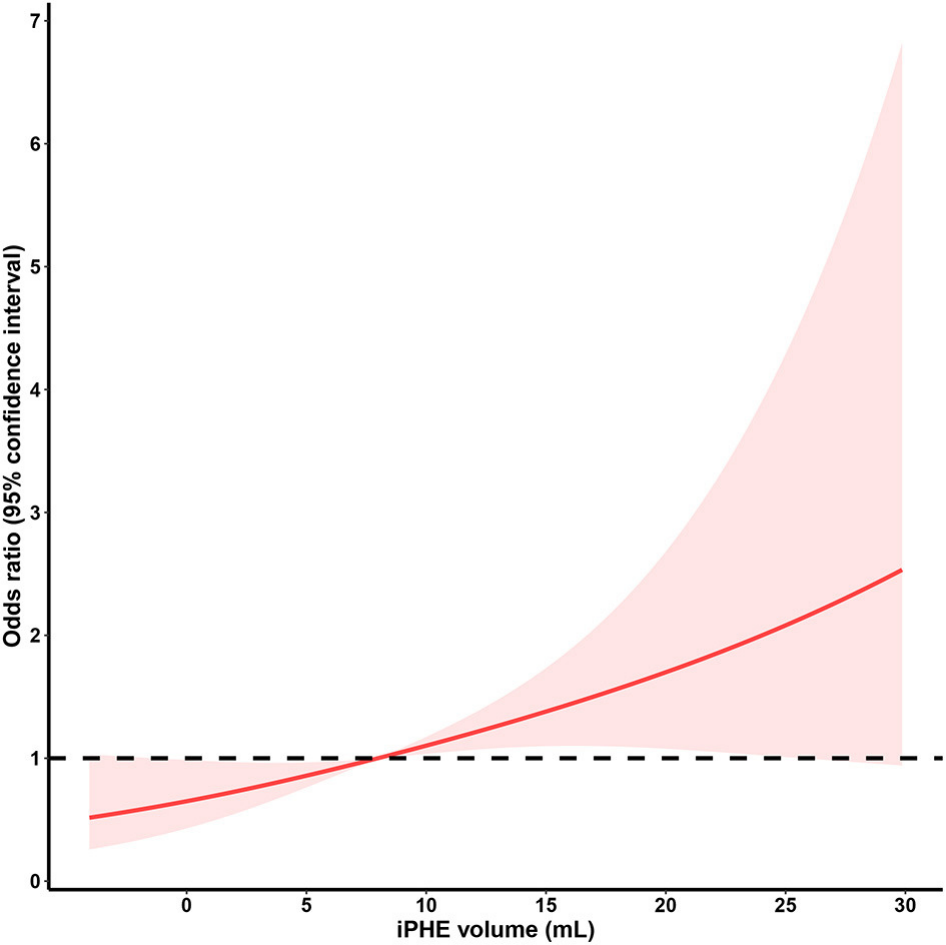
Restricted cubic spline analysis of the association between iPHE volume and poor outcome OR (The middle solid line indicates the point estimates of odds ratios and the shaded part indicate the lower and upper limits of the corresponding 95% confidence intervals. The horizontal dashed line was at odds ratio =1 (reference point: 7.98 mL). Three knots were used for the restricted cubic spline analysis).

**Figure 3:**
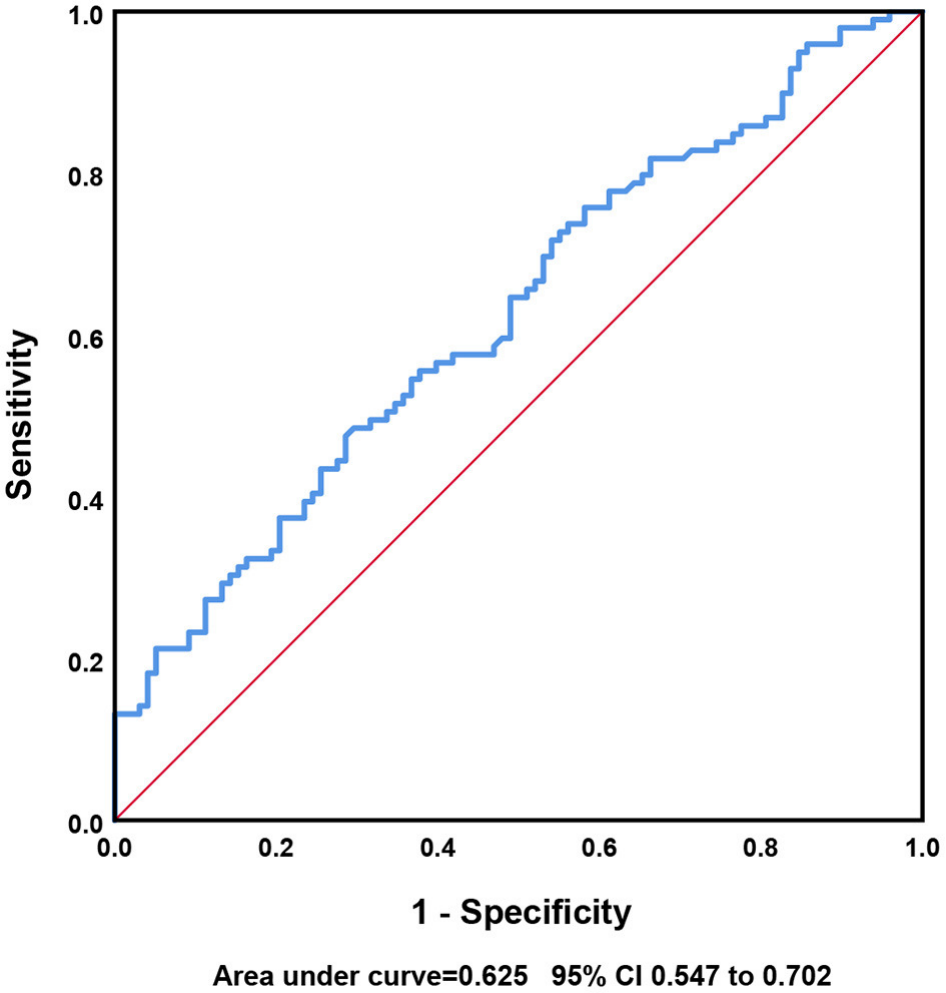
ROC analysis diagram.

iPHE volume was significantly associated with poor outcome [*P* = 0.003, odds ratio (OR) 1.049 95% confidence interval (CI) 1.016–1.082] after adjustment for hematoma volume. Analyses using the restricted cubic spline suggest a dose-response association between iPHE volume and the poor outcome OR (P for overall-association <0.001, *P* for non-linearity = 0.766, Figure 2). The ROC curves show that the best cutoff point of iPHE volume was 7.98 mL (AUC = 0.625 95% CI 0.547–0.702, Figure 3) with a specificity of 71.4% and a sensitivity of 47.5%.

### Analysis of Risk Factors for iPHE Volume

Given that iPHE volume was identified as a predictor for poor outcome, we further investigate the potential risk factors for iPHE volume. Univariate linear regression analysis showed that diabetes mellitus (*P* = 0.007, β = 10.24 95% CI 2.78–17.70), black hole sign (*P* < 0.001, β = 23.07 95% CI 11.12–35.04), and initial ICH volume (*P* = 0.023, β = 0.20 95% CI 0.03–0.38) were significantly associated with iPHE volume (Table 2). The variables with a *p*-value < 0.10 in univariate analysis were included in the multivariate analysis. The same as the former, the result of the multivariate analysis revealed a significant association between diabetes mellitus (*P* = 0.043, β = 7.66 95% CI 0.26–15.07), black hole sign (*P* = 0.002, β = 18.93 95% CI 6.84–31.02), initial ICH volume (*P* = 0.018, β = 0.20 95% CI 0.03–0.37), and iPHE volume (Table 3).

**Table 2:** Univariate linear regression analyses of iPHE volume after intracerebral hemorrhage.

**Variables**	***P*-value**	**β coefficients (95% CI)**
Age	0.704	1.029 (−4.315, 6.373)
Gender	0.167	−3.444 (−8.341, 1.452)
Symptoms at onset
Disorders of consciousness	0.574	−1.347 (−6.181, 3.433)
Seizures	0.167	−5.847 (−19.176, 7.482)
Neurological impairment	0.737	1.102 (−5.373, 7.578)
Time from onset to admission scan	0.761	−0.119 (−0.142, 0.104)
Body temperature at admission	0.331	−2.961 (−8.950, 3.028)
Admission SBP	0.925	−0.005 (−0.103, 0.094)
Hypertension	0.387	0.145 (−0.185, 0.476)
Diabetes mellitus	0.007	10.242 (2.783, 17.702)
Total leukocyte count	0.348	−0.366 (−1.137, 0.405)
Neutrophil count	0.560	−0.228 (−1.004, 0.547)
Neutrophil-to-lymphocyte ratio	0.950	−0.003 (−0.096, 0.090)
Red blood cell count	0.508	−1.457 (−5.805, 2.891)
Seralbumin	0.353	−0.347 (−1.083, 0.388)
Admission serum glucose	0.772	0.161 (−0.931, 1.252)
HCO3	0.529	−0.043 (−0.176, 0.091)
Deep vs. lobar ICH	0.697	1.029 (−4.176, 6.233)
Hemisphere ipsilateral	0.133	−3.366 (−7.766, 1.035)
Presence of IVH	0.070	−4.532 (−9.431, 0.368)
Initial ICH volume	0.023	0.200 (0.028, 0.372)
Initial PHE volume	0.691	−0.031 (−0.182, 0.121)
Obstructive hydrocephalus	0.230	−4.429 (−11.688, 2.830)
Shape categorical scales	0.964	0.045 (−1.945, 2.036)
Density categorical scales	0.238	1.842 (−1.229, 4.914)
Black hole sign	<0.001	23.078 (11.118, 35.038)
Swirl sign	0.631	2.549 (−7.899, 12.997)
Island sign	0.821	−1.427 (−13.822, 10.968)
Hematoma sedimentation level	0.876	−1.819 (−24.710, 21.072)

*IVH, intraventricular hemorrhage; SBP, systolic blood pressure; ICH, intracerebral hemorrhage; mRS, modified Rankin Scale; PHE, perihematomal edema; CI, confidence interval*.

**Table 3:** Multivariable linear regression analyses of iPHE volume after ICH.

**Variables**	***P*-value**	**β coefficients (95% CI)**	**VIF**
Black hole sign	0.002	18.93 (6.84, 31.02)	1.060
Diabetes mellitus	0.043	7.66 (0.26, 15.07)	1.058
Initial ICH volume	0.018	0.20 (0.03, 0.36)	1.005
Presence of IVH	0.208	−3.04 (−7.79, 0.21)	1.027

*IVH, Intraventricular hemorrhage; ICH, intracerebral hemorrhage; CI, confidence interval*.

### Further Study on Black Hole Sign

To further investigate whether the contribution of black hole sign to edema growth is related to hematoma expansion, we included black hole sign and hematoma expansion together in the regression analysis. The results show that the black hole sign can independently predict the increase of PHE (*P* < 0.001, β = 21.62 95% CI 10.10–33.15).

## Discussion

In this retrospective analysis, we show that the increase of PHE volume from baseline to day 3 was associated with 90-day poor outcome. Furthermore, an iPHE volume >7.98 mL may lead to poor outcomes. Initial ICH volume, black hole sign, and diabetes mellitus were significantly associated with iPHE volume.

Previous studies identify that the iPHE volume was associated with poor outcome (7, 9), so the conclusion of this study is consistent with the above findings. Presently, there is no confirmed definition of PHE expansion. In our study, an increase in PHE volume >7.98 mL from baseline to day 3 may lead to a poor outcome. Consequently, we may define an increase in PHE of more than 7.98 mL within 3 days of onset as PHE expansion. However, the AUC was only 0.625, and the sensitivity of the cutoff value was not sufficiently high; further analysis of larger cohorts is needed for defining the cutoff value.

Time from onset to baseline computed tomography (CT), baseline hematoma volume, 24-h hematoma growth, intraventricular extension, and higher neutrophil-lymphocyte ratio were thought to be independent predictors of 24-h PHE growth (13, 15). Currently, there are few studies on the predictors of iPHE volume. In the present study, we found that diabetes mellitus, black hole sign, and baseline hematoma volume were significantly associated with iPHE volume. Black hole sign was used as an imaging predictor for early hematoma growth; the presence of black hole sign may indicate bleeding of different age (16). After adjusting for hematoma expansion, the black hole sign could still independently predict the increase of PHE, whereas no such association was found for the other three imaging predictors. The underlying mechanisms need to be further explored.

It is well-known that hyperglycemia can result in impaired capillary integrity, which may theoretically lead to greater edema. However, no association between serum glucose and PHE volume was found in previous studies (22, 23). In our study, the admission serum glucose of the excluded patients was significantly higher than that of the included patients, which may be due to stress hyperglycemia caused by the more severe condition in the excluded patients. The linear regression results show that admission serum glucose was not associated with iPHE volume, but iPHE volume was significantly increased in diabetics. Considering that diabetes mellitus is a chronic metabolic disorder, the long-term blood glucose level before the onset of ICH may need more attention.

There are several limitations to this study. First, we excluded a large number of cases in order to obtain the CT scan reviewed at a specified time. Patients in the enrollment cohort had higher GCS scores, lower admission glucose, and longer time from onset to admission CT scan than those in the exclusion cohort. This suggests that our conclusions cannot be generalized to all patients with ICH but are more applicable to patients who are less acutely ill at admission and inclined to conservative treatment. However, in fact, these patients with less primary injury often need to pay more attention to the impact of secondary damage. Second, although our study collected multiple variables, residual confounding from unmeasured confounders may remain. A larger prospective multicenter cohort of Risa-MIS-ICH study has not yet completed data collection, and more data will be available to verify the conclusion of the present study.

## Conclusions

An increase of PHE volume >7.98 mL from baseline to day 3 may lead to poor outcome. Further prospective cohort studies are needed to investigate the significance of black hole sign for iPHE volume. In addition, the long-term blood glucose levels before the onset of ICH need more attention. Upon completing collection of prospective data of the Risa-MIS-ICH study, we will further validate the conclusion of the present study.

## Data Availability Statement

The raw data supporting the conclusions of this article will be made available by the authors, without undue reservation.

## Ethics Statement

The studies involving human participants were reviewed and approved by the ethics review committee of The First Affiliated Hospital of Fujian Medical University (Ethical Approval Number: MRCTA, ECFAH of FMU [2018] 082-1). The patients/participants provided their written informed consent to participate in this study.

## Author Contributions

GY contributed conception and design of the study and wrote the first draft of the manuscript. XW, QH, and RC organized the database. GY and SH performed the statistical analysis. QH and KM revised the manuscript. DK and XW supported, supervised, and designed the study. All authors contributed to critical revision of the final manuscript and approved the final version of the manuscript.

## Conflict of Interest

The authors declare that the research was conducted in the absence of any commercial or financial relationships that could be construed as a potential conflict of interest.

## Publisher's Note

All claims expressed in this article are solely those of the authors and do not necessarily represent those of their affiliated organizations, or those of the publisher, the editors and the reviewers. Any product that may be evaluated in this article, or claim that may be made by its manufacturer, is not guaranteed or endorsed by the publisher.
